# 

**Published:** 1875-05-01

**Authors:** 


					﻿No. IX.
MAY 1, 1876.
Vol. XVI.
THE
Medical Iiamiieb-
x A
mmi-fflonihli) :|oui[nal off Ifltdra! Wtitntra
EDITED BY
N. S. DAVIS, M. D., and F. H. DAVIS, M. D.
Subscription Price, Three Dollars per Annum, in advance.
Single Numbers, Fifteen Cents.
^CHICAGO.
PUBLISHED AT 65 PL RA XOO LPH STREET.
				

